# A high neutrophil-to-lymphocyte ratio is a poor prognostic factor for castration-resistant prostate cancer patients who undergo abiraterone acetate or enzalutamide treatment

**DOI:** 10.1186/s12885-020-07410-2

**Published:** 2020-09-25

**Authors:** Takashi Kawahara, Masashi Kato, Kenichi Tabata, Ippei Kojima, Hiroshi Yamada, Osamu Kamihira, Hideyasu Tsumura, Masatsugu Iwamura, Hiroji Uemura, Yasuhide Miyoshi

**Affiliations:** 1 Departments of Urology and Renal Transplantation, Yokohama City University Medical Center, Yokohama, 2320024 Japan; 2 Department of Urology, Nagoya University, Nagoya, 4668560 Japan; 3 Department of Urology, Kitasato University School of Medicine, Sagamihara, 2520375 Japan

**Keywords:** The NLR, Abiraterone acetate, Enzalutamide

## Abstract

**Background:**

Inflammatory cytokine markers, including the neutrophil-to-lymphocyte ratio (NLR), monocyte-lymphocyte ratio, and platelet-to-lymphocyte ratio, play important roles as prognostic markers in several solid malignancies, including prostate cancer. We previously reported the NLR as a poor prognostic marker in bladder cancer, upper-urothelial carcinoma, adrenocortical carcinoma, penile cancer, and prostate cancer. This study examined the importance of the NLR as a prognostic marker for castration-resistant prostate cancer (CRPC) patients who received abiraterone acetate or enzalutamide.

**Methods:**

A total of 805 prostate cancer patients developed in CRPC status were enrolled in this study. Of these patients, 449 received abiraterone acetate (ABI; 188 cases) or enzalutamide (ENZ; 261 cases) treatment, and the pre-treatment NLR values of these patients were obtained. We investigated the prognosis in those with higher and lower NLR values.

**Results:**

The median NLR was 2.90, and a receiver operating characteristics analysis suggested a candidate cut-off point of 3.02. The median overall survival (OS) was 17.3 months in the higher NLR group (≥3.02) and 27.3 months in the lower NLR group (< 3.02) (*p* < 0.0001). This trend was also observed in both the ABI and ENZ groups (ABI: 29.3 vs. 15.1 months; ENZ: NR vs. 19.5 months; *p* < 0.0001 and < 0.0001, respectively). A multivariate analysis revealed that a higher NLR was an independent risk factor. The NLR value was thus shown to be correlated with the prostate cancer progression.

**Conclusions:**

A higher NLR was associated with a poorer OS for CRPC patients who received ABI or ENZ. The NLR was positively correlated with prostate cancer progression.

## Background

Some inflammatory cytokine markers, including the neutrophil-to-lymphocyte ratio (NLR), monocyte-lymphocyte ratio (MLR), and platelet-to-lymphocyte ratio, play important roles as prognostic markers in certain solid malignancies, including prostate cancer [1]. We previously reported the NLR to be a poor prognostic marker in bladder cancer, upper-urothelial carcinoma, penile cancer, and prostate cancer [2–9]. In prostate cancer, the higher NLR group showed a higher incidence of prostate cancer among patients whose PSA was 4–10 ng/ml. A higher NLR was also associated with a poorer overall survival (OS) in metastatic hormone-sensitive prostate cancer (mHSPC) and castration-resistant prostate cancer (CRPC) patients who underwent docetaxel or cabazitaxel systemic chemotherapy [3–7, 10, 11]. However, there have been no reports on the NLR and prognosis of CRPC patients who received abiraterone acetate (ABI) and enzalutamide (ENZ). A recent clinical trial revealed the efficacy of ABI, ENZ, Radium-223 (Ra-223), and cabazitaxel in addition to docetaxel chemotherapy in metastatic castration-resistant prostate cancer (mCPRC) patients [12–14]. In the next few years, poly (ADP-ribose) polymerase (PARP) inhibitors and immune-checkpoint inhibitors are expected to be used in clinical practice to similar ends [15, 16]. With widespread medication choices now available, clinicians should take care to select the most appropriate medicine in order not to lose their chance to administer the best therapy possible to a patient. Predicting the prognosis is thus important, because a lack of biomarker to predict the efficacy of each mCRPC treatment. NLR is easily calculated using complete blood cell counts (CBCs) which was widely measured in daily clinical practices. Thus, previous data was easily obtained in each patients in each prostate cancer progression stage. And no additional cost is required.

The present study examined the utility of the NLR as a prognostic marker for CRPC patients who received ABI and/or ENZ.

## Methods

### Patients

A total of 805 prostate cancer patients who developed CRPC in Yokohama City University, Kitasato University, Nagoya University, and affiliated hospitals were enrolled in this study. Of these patients, 449 received ABI or ENZ, and the pre-treatment NLR values of these patients were obtained (Table 1). All patients received initial androgen deprivation therapy or combined androgen blockade treatment and were refractory to each treatment. The definition of CRPC was set by the Prostate Cancer Working Group 2 [17]. The patients who received both ABI and ENZ were classified as the first-line treatment group.

**Table 1:** Patients’ characteristics

	n (%) or median (mean +/− SD)		
Variables	ABI (n:188)	ENZ(n:261)	*p* value
Age			
> 75.6 years	105 (55.9%)	120 (46.0%)	0.039
≤ 75.6 years	83 (44.1%9	141 (54.0%)	
Initial distant Metastasis			
Yes	87 (46.3%)	157 (60.2%)	0.248
No	65 (34.6%)	99 (37.9%)	
Unknown	36 (19.1%)	5 (1.9%)	
Initial PSA (ng/mL)	110 (616.2 +/− 1467.7)	100 (648.1 +/− 1534.9)	< 0.001
Gleason Score			
≥ 8	93 (49.5%)	121 (46.4%)	0.515
< 8	95 (50.5%)	140 (53.6%)	
Pre ABI/ENZ PSA (ng/mL)	26.0 (189.8 +/− 595.6)	38.8 (233.3 +/− 737.5)	< 0.001
Pre DOC Treatment			
Yes	61 (32.4%)	145 (55.6%)	< 0.001
No	127 (67.6%)	116 (44.4%)	
ALP			
> 274 IU/L	92 (48.9%)	119 (45.6%)	0.484
≤ 274 IU/L	96 (51.1%)	142 (54.4%)	
LDH			
> 220 IU/L	96 (51.1%)	122 (46.7%)	0.366
≤ 220 IU/L	92 (48.9%)	139 (53.3%)	

*PSA* Prostate-specific antigen, *CRPC* Castration-resistant prostate cancer, *DOC* Docetaxel

*ABI* Abiraterone, *ENZ* Enzalutamide, *ALP* Alkaline phosphatase, *LDH* Lactate dehydrogenase

This study was approved by the Institutional Review Board of Yokohama City University Medical Center (Yokohama, Japan) (IRB No. D1603004). Written informed consent was waived due to the retrospective observational nature of the study, and all methods complied with the Declaration of Helsinki.

### Assessment of the NLR

The pretreatment NLR was calculated using the complete blood cell count (CBC). To exclude any effect on the NLR, cases with C-reactive protein elevation or infection were excluded, as in our previous study [9]. Candidate cut-off points of the NLR were determined by the area under the receiver operator characteristic curve (AUROC).

### Prostate cancer progression and the NLR transition

To examine the NLR transition in prostate cancer status, we compared our previous reports as follows (Table 2): 1) 811 patients whose PSA was 4 to 10 ng/mL, including 357 localized prostate cancer and 453 pathological confirmed non-prostate cancer patients [3]; 2) 48 metastatic hormone-naïve prostate cancer (mHNPC) patients [7]; 3) 47 CRPC patients resistant to docetaxel and who received cabazitaxel chemotherapy [10]; and 4) this cohort, including 243 pre-docetaxel and 206 post-docetaxel patients.

**Table 2:** Summarize of pretreatment NLR in each prostate cancer status

Treatment	n	Journal
Pre and post DOC treatment	449	Current Study
Pre CBZ treatment	47	Uemura and Kawahara et al. Biomed Res Int. 2017
Initial metastatic prostate cancer	48	Kawahara and Yokomizo et al. BMC Cancer 2016
PSA 4 to 10 ng/mL	810	Kawahara and Fukui et al. Oncotarget 2015

*DOC* Docetaxel, *CBZ* Cabazitaxel, *PSA* Prostate-specific antigen

### Statistical analyses

The patients’ characteristics and preoperative factors were analyzed by the Mann-Whitney *U* and chi-square tests, using the Graph Pad Prism software program (Graph Pad Software, La Jolla, CA, USA). Candidate cut-off points were identified using the AUROC. We set the endpoint as death 3 years from ENZ/ABI installation. The candidate cut-off points were determined using Younden’s index. The survival duration was defined as the time between the dates of initial ABI/ENZ installation and the time of death. A log-rank test was performed for comparisons between the higher and lower NLR groups. Univariate and multivariate analysis was performed to determine whether or not the NLR was an independent prognostic factor. Multivariate analysis was performed using the clinical important variables including ENZ vs ABI, metastasis vs non-metastasis, Gleason score ≧8 vs < 8, age, pre DOC treatment, NLR, ALP, and LDH. We also performed multivariate analysis categorized and continuous values. *P* values of < 0.05 were considered to indicate statistical significance.

## Results

**Fig. 1:**
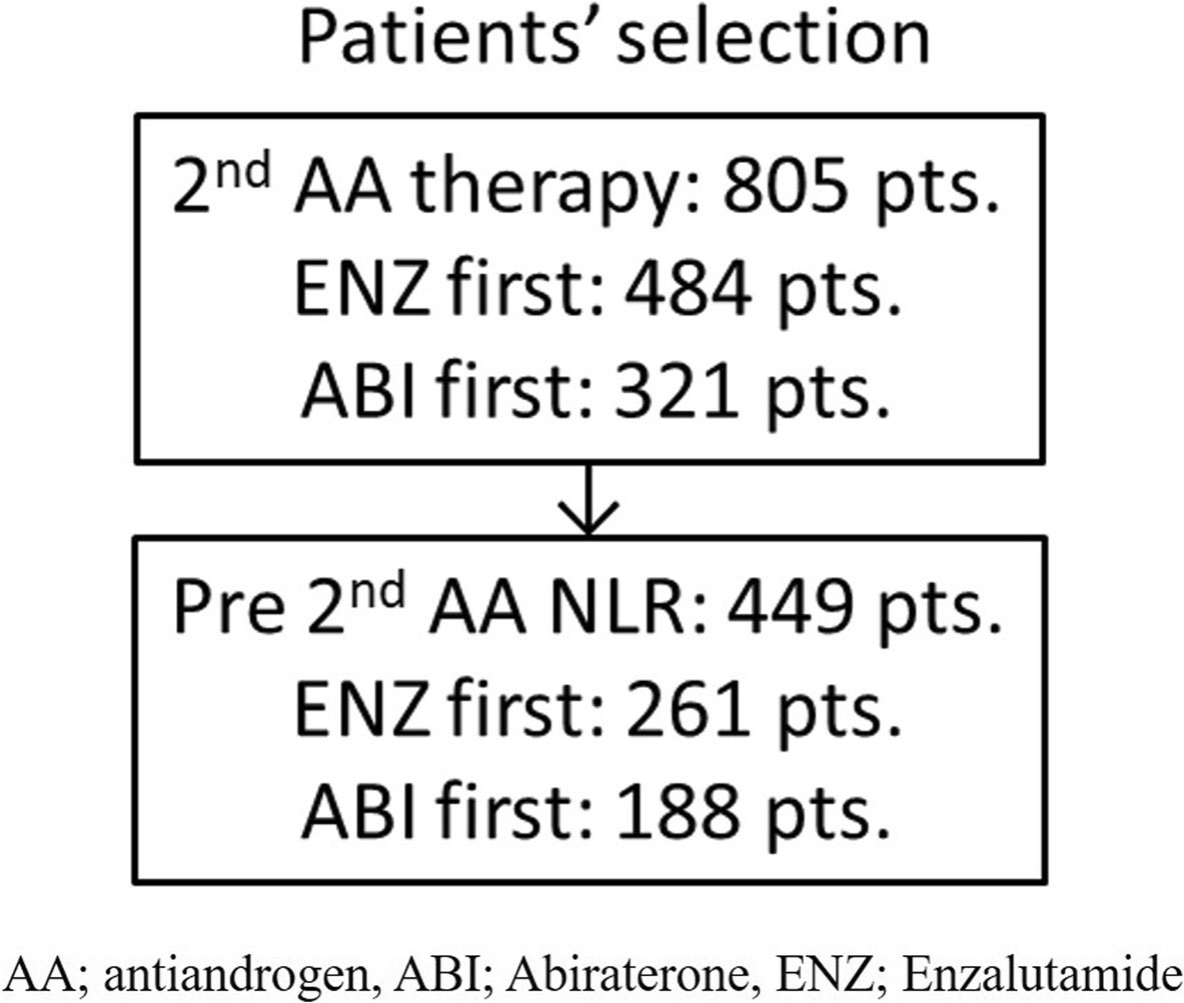
Patient selection

A total of 805 CRPC patients received ABI or ENZ treatment, and 449 of them had available NLR data (Fig. 1). A total of 261 (58.1%) cases underwent ENZ first, and 188 (41.9%) underwent ABI first. Of these patients, 206 (45.9%) received docetaxel chemotherapy for CRPC preoperatively. None of the patients received upfront docetaxel or upfront abiraterone therapy for hormone-naïve status. The median (mean ± SD) follow-up duration was 12.0 (11.8 ± 7.1) months.

**Fig. 2:**
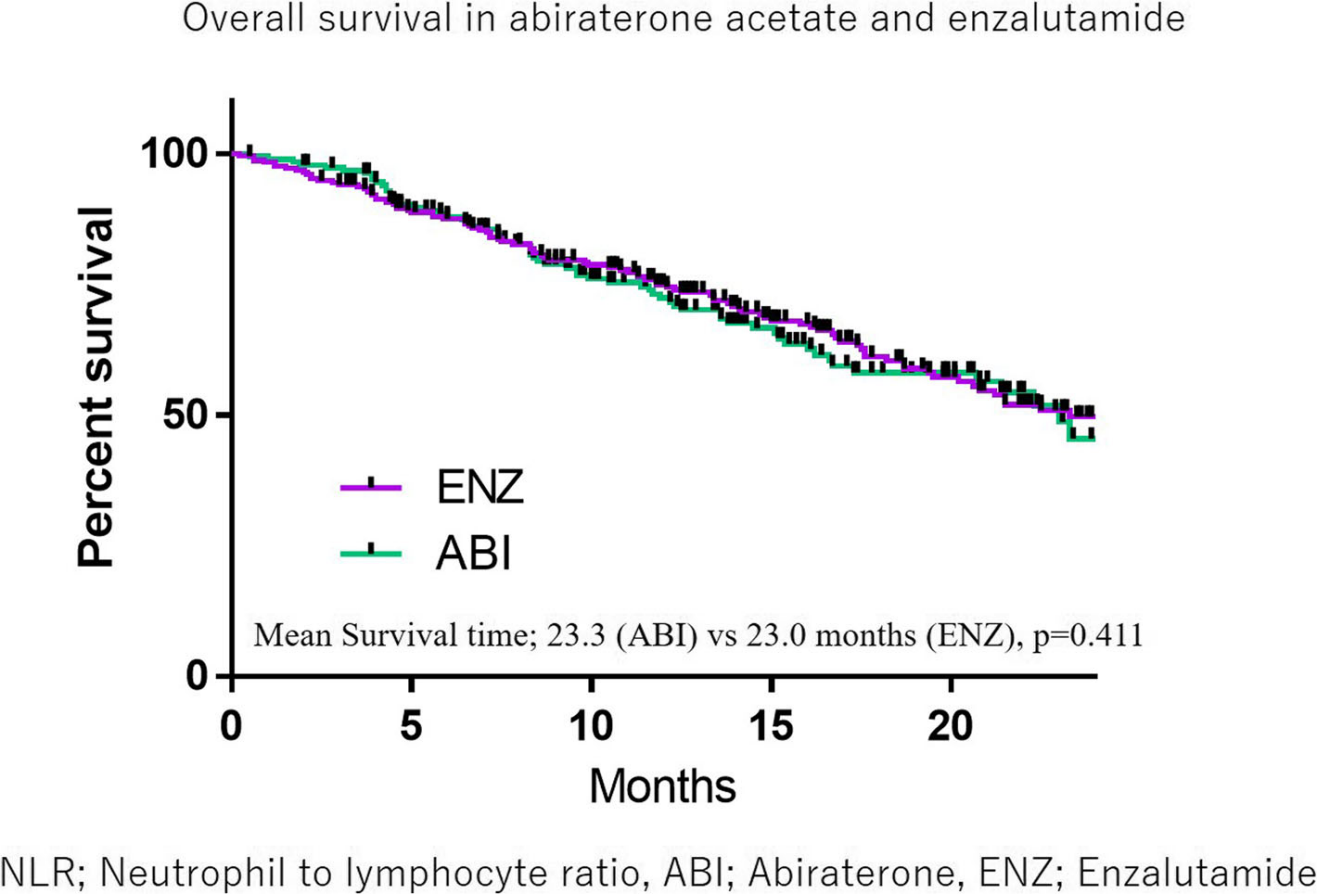
Receiver operator characteristic curve

**Fig. 3:**
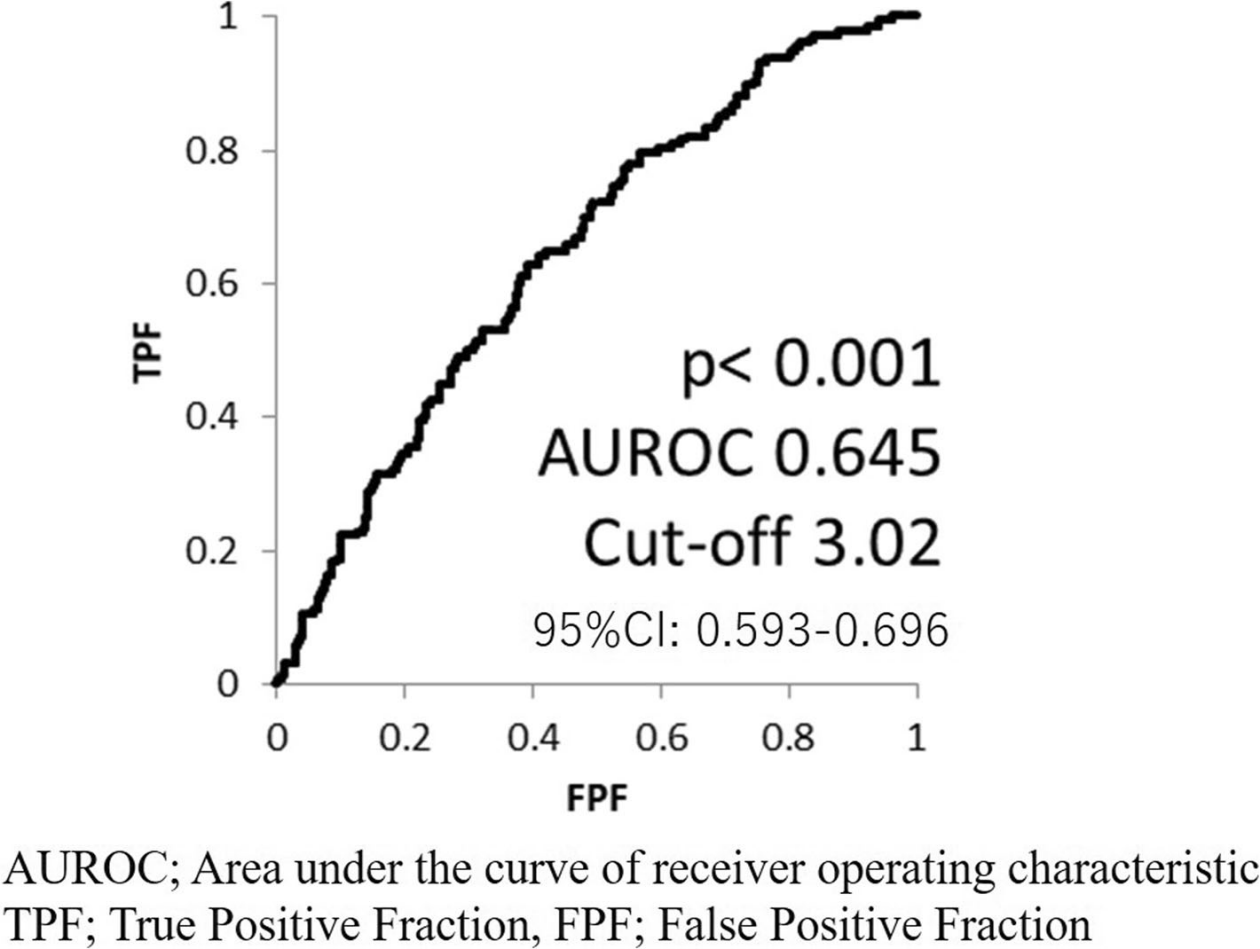
The overall survival in abiraterone acetate and enzalutamide

**Fig. 4:**
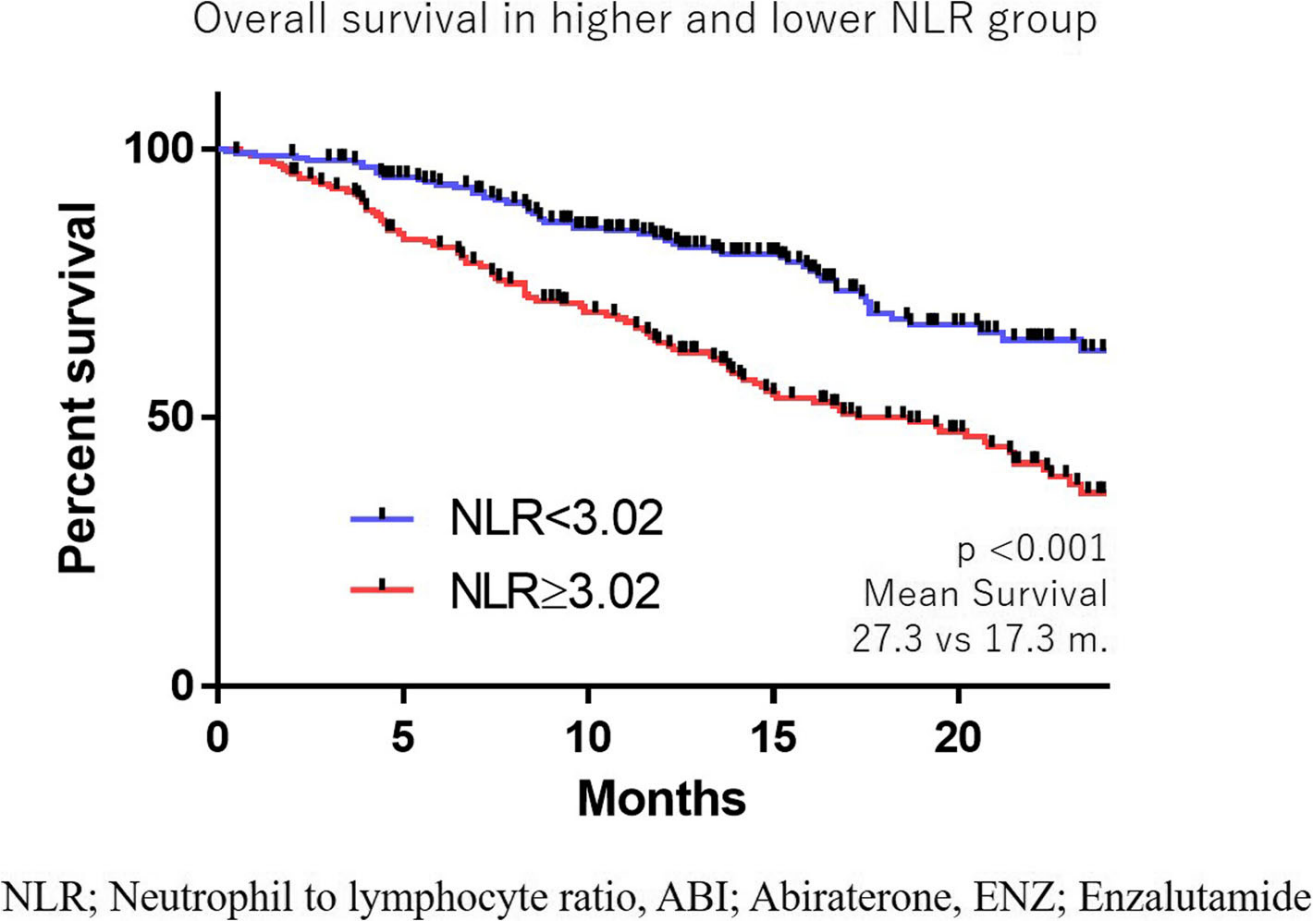
The overall survival in the higher and lower NLR groups

**Fig. 5:**
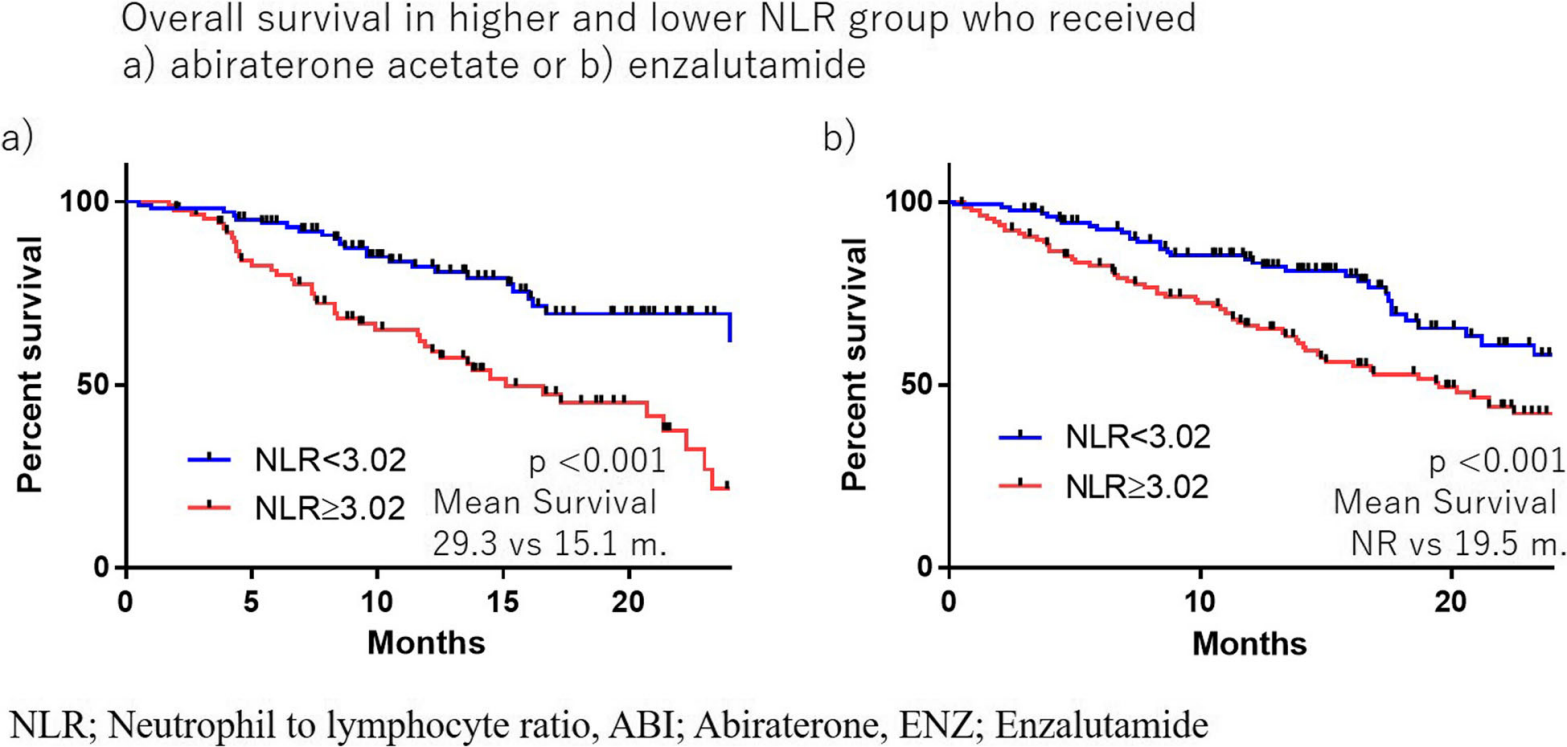
The overall survival in the higher and lower NLR groups who received a) abiraterone acetate or b) enzalutamide

There were no marked differences in the OS between the ABI and ENZ groups (23.3 vs. 23.0 months, *p* = 0.411) (Fig. 2). The median NLR was 2.90, and the ROC suggested a candidate cut-off point of 3.02 (Fig. 3). The median OS was 17.3 months in the higher NLR group (≥3.02) and 27.3 months in the lower NLR group (< 3.02) (*p* < 0.0001) (Fig. 4). This trend was also observed in the ABI and ENZ groups (ABI: 29.3 vs. 15.1 months, ENZ: not reached vs. 19.5 months, *p* < 0.0001 and < 0.0001, respectively) (Fig. 5). A multivariate analysis showed that a higher NLR (using the cut-off points based on the ROC), pre-docetaxel status, higher ALP (median value or more), and higher LDH (median value or more) were independent risk factors (Table 3). We also performed a multivariate analysis using continuous age, NLR, ALP, and LDH values and showed that the higher NLR, higher ALP, and higher LDH were independent risk factors for a poor prognosis (Supplementary Table 1).

**Table 3:** Univariate and multivariate analyses of factors associated with overall survival

Variables	Univariate				Multivariate			
	HR	95%CI		*p* value	HR	95%CI		*p* value
		Lower	Upper			Lower	Upper	
ENZ vs ABI	1.280	0.873	1.884	0.205	1.105	0.810	1.507	0.529
Metastasis vs non-metastasis	1.200	0.835	1.719	0.328	1.412	1.011	1.970	0.043
Gleason score ≥ 8 vs Gleason score < 8	0.810	0.566	1.148	0.232	1.004	0.731	1.379	0.981
Age ≥ 75.6 (median) vs < 75.6	1.100	0.770	1.571	0.601	0.988	0.729	1.338	0.938
DOC treatment vs non-DOC treatment	2.070	1.410	3.045	< 0.001	2.160	1.571	2.970	< 0.001
NLR > 3.02 vs ≤3.02	2.070	1.094	2.300	0.015	2.115	1.540	2.906	< 0.001
ALP > 274 (median) vs ≤274	1.580	1.295	2.760	< 0.001	2.189	1.579	3.035	< 0.001
LDH > 220 (median) vs ≤220	1.790	1.234	2.597	0.002	2.284	1.647	3.168	< 0.001

*HR* Hazard ratio, *CI* Confidential interval, *ABI* Abiraterone, *ENZ* Enzalutamide, *DOC* Docetaxel

*NLR* Neutrophil to lymphocyte ratio, *ALP* Alkaline phosphatase, *LDH* Lactate dehydrogenas

**Fig. 6:**
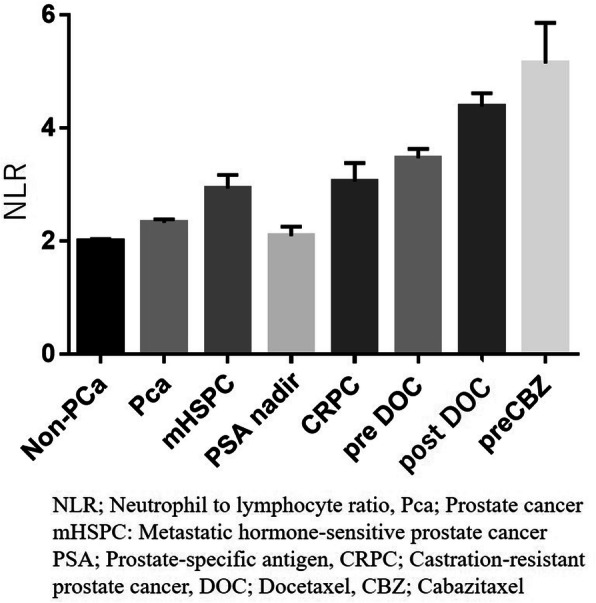
The NLR changes by prostate cancer status. Mean +/− SEM

The NLR value was found to be correlated with prostate cancer progression, gradually increasing as pathological non-adenocarcinoma with PSA 4 to 10 ng/mL, pathological confirmed adenocarcinoma with PSA 4 to 10 ng/mL, mHNPC, pre-docetaxel CRPC, post-docetaxel CRPC, and docetaxel resistant pre-cabzitaxel patients. Interestingly, the NLR at the time of the PSA nadir was lower than mHSPC or CRPC status (Fig. 6).

## Discussion

This study investigated the importance of the NLR as a prognostic factor for CRPC patients who receive first-line androgen replacement therapy (ART), including ENZ or ABI. The NLR has been reported as a prognostic factor for several solid malignancies, including prostate cancer. Our previous reports showed that the NLR played important role as whether prostate cancer or not in patients with a serum PSA level of 4 to 10 ng/mL, or as a prognostic factor in mHNPC [3, 14]. In the present study, we further evaluated the value of the NLR for predicting prostate cancer progression. The NLR was found to be correlated with prostate cancer progression as low value in PSA between 4 to 10 or prostate cancer patients received radical prostatectomy, or the situation the prostate cancer patients whose PSA were nadir after ADT [18]. In contrast, the NLR gradually increased in cases of pre-docetaxel CRPC as well as in patients with a post-docetaxel status. Based on these findings, the NLR might not be simply a prognostic factor but also a tumor-related marker reflecting prostate cancer aggressiveness.

The details concerning the mechanism underlying the association of the NLR and prostate cancer aggressiveness are unclear. Some inflammatory markers, including the NLR, MLR, total lymphocyte count (TLC), and psoas muscle index (PMI), have been reported to be poor prognostic markers in solid malignancies [1]. These markers are increased under conditions of a reduced lymphocyte count [19]. No marked differences were noted in the OS between the ABI and ENZ groups in our study. Kim et al. also found no marked differences in the OS for CRPC patients who used ENZ-ABI sequence or ABI-ENZ sequence [20]. Our study also showed similar results to real-world clinical practice. Tumor markers to predict the efficacy of ABI or ENZ are needed, but our results indicated that a high NLR was associated with a poor prognosis in both ABI and ENZ groups.

A previous study showed that the NLR was higher in cases of mHSPC than in localized prostate cancer, and its increase correlated with treatment resistance in cases of CRPC [7]. Furthermore, the NLR was decreased at the time of the PSA nadir. Most previous studies showed the NLR to be a prognostic factor, but the present study revealed a correlation between the NLR and prostate cancer progression [21–24]. The NLR might thus reflect the current prostate cancer aggressiveness.

Several limitations associated with the present study warrant mention. First, this was a retrospective observation study, and quite a few cases were excluded because pre-ABI or pre-ENZ CBCs had not been collected. However, despite this limitation, this was the first study to evaluate the pre-ABI/pre-ENZ NLR in a multicentral study. Second, this study did not reveal the mechanisms underlying the association between the NLR and prostate cancer progression. Further studies will be needed to confirm this point using the same patient cohort with continuous follow-up CBCs. Third, this study was unable to determine the power of NLR as a prognostic factor for cancer progression. Both a high NLR using the cut-off point (> 3.02) and continuous number of NLR values were independent risk factors for a poor prognosis. On the other hand, the HR was differed using these different methods. And also we could not evaluated the power of poor prognosis comparing to the other previous reports. Further studies will be needed to determine good candidate cut-off points for daily clinical practice. The last one is that these data was obtained from each institutional data subsets. Thus, central testing is needed for future study to confirmed no differences between each institutions.

## Conclusion

A higher NLR was associated with a poor OS in CRPC patients who received ABI or ENZ. The NLR was positively correlated with prostate cancer progression.

## Supplementary information

**Additional file 1.**

## Data Availability

Due to ethical restrictions, the raw data underlying this paper is available upon request from the corresponding author.

## Abbreviations

NLR: neutrophil-to-lymphocyte ratio
CRPC: castration-resistant prostate cancer
ABI: abiraterone acetate
ENZ: enzalutamide
OS: overall survival
MNLR: monocyte-lymphocyte ratio
mHSPC: metastatic hormone-sensitive prostate cancer
CBC: complete blood cell count
AUROC: area under the receiver operator characteristic curve
