# Supplementary material for: A high neutrophil-to-lymphocyte ratio is a poor prognostic factor for castration-resistant prostate cancer patients who undergo abiraterone acetate or enzalutamide treatment
Source: BMC Cancer. 2020 Sep 25;20:919. doi: 10.1186/s12885-020-07410-2 (PMC7519532; doi:10.1186/s12885-020-07410-2)
Supplement: Supplementary file 1 — Additional file 1. [file 12885_2020_7410_MOESM1_ESM.docx]

| Supplementary Table 1: Univariate and multivariate analyses of factors associated with overall survival | | | | | | | | |
| --- | --- | --- | --- | --- | --- | --- | --- | --- |
| Variables | Univariate | | | | Multivariate | | | |
|  | HR | 95%CI | | p value | HR | 95%CI | | p value |
|  |  | Lower | Upper |  |  | Lower | Upper |  |
| ENZ vs ABI | 1.105 | 0.810 | 1.507 | 0.529 | 0.853 | 0.511 | 1.423 | 0.542 |
| Metastasis vs non-metastasis | 1.412 | 1.011 | 1.970 | 0.043 | 0.897 | 0.528 | 1.522 | 0.687 |
| Gleason score ≥8 vs Gleason score<8 | 1.004 | 0.731 | 1.379 | 0.981 | 0.476 | 0.276 | 0.820 | 0.007 |
| Age (continuous value) | 0.983 | 0.952 | 1.015 | 0.290 | 1.029 | 0.992 | 1.066 | 0.127 |
| DOC treatment vs non-DOC treatment | 2.160 | 1.571 | 2.970 | 0.000 | 1.768 | 1.027 | 3.044 | 0.040 |
| NLR (continuous value) | 1.105 | 1.052 | 1.162 | 0.000 | 1.123 | 1.063 | 1.187 | <0.001 |
| ALP (continuous value) | 1.000 | 1.000 | 1.001 | 0.000 | 1.000 | 1.000 | 1.001 | 0.001 |
| LDH (continuous value) | 1.002 | 1.001 | 1.002 | 0.000 | 1.002 | 1.001 | 1.003 | <0.001 |
| HR: Hazard ratio, CI; Confidential interval, ABI; Abiraterone, ENZ; Enzalutamide, DOC; Docetaxel | | | | | | | | |
| NLR; Neutrophil to lymphocyte ratio, ALP; Alkaline phosphatase, LDH; Lactate dehydrogenase | | | | | | | | |
